# Appetite for self-destruction: suicidal biting as a nest defense strategy in *Trigona* stingless bees

**DOI:** 10.1007/s00265-014-1840-6

**Published:** 2014-11-08

**Authors:** Kyle Shackleton, Hasan Al Toufailia, Nicholas J. Balfour, Fabio S. Nascimento, Denise A. Alves, Francis L. W. Ratnieks

**Affiliations:** 1 Laboratory of Apiculture and Social Insects, Department of Life Sciences, University of Sussex, Brighton, BN1 9QG UK; 2 Departamento de Biologia, Faculdade de Filosofia Ciências e Letras de Ribeirão Preto, Universidade de São Paulo, Av. Bandeirantes, Ribeirão Preto, 14040-901 Brazil; 3 Departamento de Entomologia e Acarologia, Escola Superior de Agricultura “Luiz de Queiroz”, Universidade de São Paulo, Av. Pádua Dias 11, Piracicaba, São Paulo 13418-900 Brazil

**Keywords:** Self-destructive behavior, Colony defense, Self-sacrifice, Nest guards, Stingless bees, *Trigona*

## Abstract

Self-sacrificial behavior represents an extreme and relatively uncommon form of altruism in worker insects. It can occur, however, when inclusive fitness benefits are high, such as when defending the nest. We studied nest defense behaviors in stingless bees, which live in eusocial colonies subject to predation. We introduced a target flag to nest entrances to elicit defensive responses and quantified four measures of defensivity in 12 stingless bee species in São Paulo State, Brazil. These included three *Trigona* species, which are locally known for their aggression. Species varied significantly in their attack probability (cross species range = 0–1, *P* < 0.001), attack latency (7.0–23.5 s, *P* = 0.002), biting duration of individual bees (3.5–508.7 s, *P* < 0.001), and number of attackers (1.0–10.8, *P* < 0.001). A “suicide” bioassay on the six most aggressive species determined the proportion of workers willing to suffer fatal damage rather than disengage from an intruder. All six species had at least some suicidal individuals (7–83 %, *P* < 0.001), reaching 83 % in *Trigona hyalinata*. Biting pain was positively correlated with an index of overall aggression (*P* = 0.002). Microscopic examination revealed that all three *Trigona* species had five sharp teeth per mandible, a possible defensive adaptation and cause of increased pain. Suicidal defense via biting is a new example of self-sacrificial altruism and has both parallels and differences with other self-sacrificial worker insects, such as the honey bee. Our results indicate that suicidal biting may be a widespread defense strategy in stingless bees, but it is not universal.

## Introduction

Behaviors enhancing self-preservation, such as predator defense, are fundamental to survival (Alock 2005). Similarly, parental defense of offspring is widespread despite the increased risk of parental mortality, as it increases defender’s total reproduction (Andersson et al. 1980; Klemperer 1982; Sefc et al. 2008; Nazareth and Machado 2010). Social insect workers, which typically have no direct reproduction, use a variety of strategies to defend their nests against predators. In extreme cases, this involves the self-sacrifice of defenders (Shorter and Rueppell 2012). Social insect nests are worth defending as they contain not only offspring (brood) but also the reproductive individuals, food stores, and nesting material, while the nest itself is often a valuable resource (Seeley 1985; Roubik 2006). Natural selection will favor defensive self-sacrifice in worker insects if it increases their inclusive fitness more than non-suicidal defensive strategies.

Suicidal defense has evolved multiple times in social insect workers and takes various forms. Sting autotomy, which is well known in the honey bee (*Apis mellifera*), involves the self-amputation of the sting apparatus from the body. This increases venom delivery and releases alarm pheromone and the apparatus can continue to pulsate long after the stinging event (Hermann 1971; Burrell and Smith 1995). Autothysis, the rupturing of the body wall to release defensive chemicals, is known in ants (e.g., *Camponotus* spp., Maschwitz and Maschwitz 1974; Davidson et al. 2012) and termites (e.g., *Globitermes* spp., Bordereau et al. 1997). A similar mechanism has been described in aphids, which produce a sticky secretion causing the defending aphid to adhere to the predator, thereby immobilizing it (Uematsu et al. 2010). All of these strategies combine a behavioral component with morphological adaptations which inevitably cause mortality in the defending workers. Worker self-sacrifice, however, need not require morphological specializations. For example, worker *Forelius pusillus* ants have a form of pre-emptive suicidal defensive behavior (Tofilski et al. 2008). Workers seal their nest entrances from the outside in the evening, resulting in most dying before the entrance is reopened from the inside in the morning.

Stingless bees (Meliponinae) comprise many hundred described species, are found worldwide in the tropics, are closely related to honey bees, and live in perennial eusocial colonies of c. 100–100,000 workers (Michener 2000; Roubik 2006). As their name suggests, stingless bees are unable to sting as the stinger is vestigial (Michener 2000). However, they still face predation at the nest from many sources ranging from mammals to nest-robbing bees (Wille 1983; Suka and Inoue 1993; Roubik 2006). Defense, therefore, is important for colony survival. Despite lacking a sting, stingless bees possess numerous defensive mechanisms including biting, harassment, caustic chemicals, alarm pheromones, and hovering guards (Kerr and de Lello 1962; van Zweden et al. 2011). Observations of *Trigona* spp. stingless bees in Brazil indicate that humans standing in the vicinity of nests are invariably attacked (FR, personal observations). These *Trigona* workers give a painful and persistent bite, are difficult to dislodge, and frequently die in the attack. Buchwald and Breed (2005) also noted grappling and biting behavior in *Trigona* in conflicts with other bees, where individuals would refuse to disengage from each other resulting in death. Individuals inevitably die in colony defense, but if biting workers are more willing to die than disengage from an intruder, it would constitute suicidal behavior.

Suicidal biting in stingless bees has not been formally reported in the literature (Shorter and Rueppell 2012). If it does occur, it would be a novel form of self-destructive behavior. This study aimed to determine whether the intense biting that we have casually experienced in *Trigona* bees is so extreme as to justify being considered a form of suicidal defense. We carried out a field study of three *Trigona* species in São Paulo State, Brazil, that personal experience had indicated are candidates. We studied nine further stingless bee species to put the *Trigona* results in a wider context. Our results show that workers of all three *Trigona* and three of the other nine species bit a target “intruder” so persistently and tenaciously that a significant proportion suffered fatal physical damage.

## Methods

### Study sites and species

The study was conducted in São Paulo State, Brazil, at two locations. Most stingless bee colonies were located on the campus of the University of São Paulo at Ribeirão Preto. The remainder were c. 50 km away at Fazenda Aretuzina, a farm near the town of São Simão dedicated to wildlife conservation and stingless bee research (Table 1). All field data were collected between 0800 and 1735 hours on sunny days in March 2014 at temperatures of 25–35 °C.

**Table 1:** List of the 12 stingless bee study species, colony locations, number of colonies used, number of flag tests performed, and number of bees biting the flags

Species	Colony locations	Bee nesting sites	Typical colony size (no. foragers)	Number of colonies	Number of flag tests	Number of biting bees
*Trigona hyalinata*	USP, FA	Buildings, trees	Large	5	28	255
*Trigona fuscipennis*	USP	Trees	Large	2	14	104
*Trigona spinipes*	USP	Trees	Large	2	14	146
*Partamona helleri*	USP	Buildings, trees, apiaries	Small	4	20	129
*Scaptotrigona depilis*	USP	Apiaries	Medium	5	30	109
*Tetragona clavipes*	USP	Trees, apiaries	Medium	7	30	68
*Tetragonisca angustula*	USP	Apiaries	Small	10	40	38
*Frieseomelitta varia*	USP	Apiaries	Small	5	30	10
*Melipona scutellaris*	USP	Apiaries	Very small	5	30	0
*Melipona quadrifasciata*	USP	Apiaries	Very small	5	30	6
*Melipona rufiventris*	FA	Apiaries	Very small	5	15	0
*Leurotrigona muelleri*	USP	Tree stumps, apiaries	Very small	4	20	0

*USP* University of São Paulo at Ribeirão Preto, *FA* Fazenda Aretuzina near São Simão

A total of 12 species were studied (Table 1). We aimed to study at least three colonies of each species, but for two species, *Trigona fuscipennis* and *Trigona spinipes*, we were only able to locate two colonies of each (Table 1). Previous experience indicated that colonies of *Tetragonisca angustula* were highly variable in their aggression toward perceived threats. Therefore, 10 colonies of this species were studied. The majority of colonies were kept in hives within apiaries, but some were wild and nesting in trees or on buildings (Table 1). All hive-dwelling colonies had modified their nest entrances using wax and resin to construct their “natural” entrance structures.

### Defensivity bioassays

We performed two field bioassays to quantify aggression and suicidal behavior. To induce bee colonies into attacking, we used black felt flags, 10 × 10 cm mounted on poles, as used in previous research on honey bee defensive behavior (Hunt et al. 1998). In a flag test, the flag was waved within 5 cm from a colony entrance for a period of 1 min or until the bees attacked, whichever was sooner. An attack was defined as one or more bees leaving the nest entrance, landing on the flag, and proceeding to bite. If an attack occurred, we recorded the time at which it began (latency) following the start of the flag test and then carefully removed the flag to a distance of 5–10 m from the colony. We then recorded the number of bees biting and the duration of attack for each bee. A particular bee’s attack was deemed to have ended when the bee left the vicinity of the flag (bees would occasionally leave the flag but return seconds later). Additionally we calculated “overall aggression” as a descriptive measure defined as $\frac{PND}{L}$, where *P* = probability of attack, *N* = number of bees, *D* = log_10_ attack duration, and *L* = log_10_ latency. Fresh flags were used following each attack to exclude the effect of any previously deposited alarm pheromones. We tried to study the same colony no more than twice on a single day, but in a few cases, this was not possible.

A suicide bioassay was performed only on species which, in the flag tests, had a probability of attack >0.5 and a mean attack duration >15 s. Bees attacked a flag as before. The flag was then removed to a distance of 20 m from the nest. Biting bees were subjected to two levels of the bioassay in order to test their degree of self-sacrifice. Firstly, bees were brushed for 5 s using a 5-mm-width paintbrush which caused no physical harm. Secondly, remaining bees had their wings on both sides clamped using a pair of forceps. Forceps have been used previously to induce suicidal responses in *Camponotus* ants (Maschwitz and Maschwitz 1974). Bees were pulled until they either let go of the flag and could subsequently fly away when released or suffered damage to the wing to the point that they could no longer fly when released. The damage usually consisted of large portions of the wing membrane being removed or a whole wing separating at the thorax. Occasionally, however, the bee’s body would separate between the first and second thoracic segments leaving the mandibles, head, and first thoracic segment clamped to the flag. Since these bees could no longer fly and return to their nests, they were deemed to have suffered fatal damage and therefore were self-sacrificing. Although the flag material was not an exact representation of any particular predator, we simply wanted to give the bees a choice between disengaging and continuing to bite the flag. We repeated this for 30 bees of each species (Table 1).

### Biting pain

In order to characterize the degree of pain caused by the bites of each species, we allowed entrance guards to bite us on the forearm. We then ranked species into distinct pain categories on a scale of 0–5 similar to that of Schmidt et al. (1983), where 0 = could not be induced to bite, 1 = biting was visible but could not pinch skin, 2 = able to pinch skin but caused no pain, 3 = very mild pain, 4 = moderate pain, and 5 = sharper unpleasant pain and capable of breaking skin if persistent. All authors were subjected to biting bees of each species and agreed upon their ranking. This was a subjective, non-linear scale, but we simply wanted to describe some bees as causing more pain than others.

### Mandibular teeth

Using a stereomicroscope, we photographed the mandibles of each study species in order to identify any characteristics which might aid in defense such as size and teeth.

### Controlling for colony size

We wanted to allow for colony size in our analysis so we used incoming forager traffic as a proxy (Couvillon et al. 2008a). Each colony in the study had its nest entrance video-recorded for a period of 3 min between 0900–1200 hours when foraging activity was high. The number of foragers returning to the colony was then counted from the video.

### Statistical analysis

For the flag test bioassay, we used mixed-effects models to fit attack probability (binomial error structure), number of bees per attack (Poisson error structure), attack latency, and attack duration (both log_10_-transformed) as response variables. Flag nested within colony was fitted as a random effect for attack duration, as we measured duration for multiple bees per flag and sampled each colony with multiple flags. Colony was fitted as a random effect for the other response variables. We also controlled for time of day, forager traffic, and attack number, as it is possible that the more attacks a colony receives, the more aggressive it may become (Couvillon et al. 2008b). The maximum models were fitted then simplified to the minimum adequate models through backward elimination of non-significant terms and model comparison using ANOVA. For the suicide bioassay, we fitted the degree of self-sacrifice as the response in a mixed-effects model (alive or dead, binomial errors) with species as the explanatory variable and colony as a random effect. We present test statistics and *P* values of our minimum adequate models compared to the null models using ANOVA. Finally, we used Spearman’s rank correlation to look for any association between pain and overall aggression, where each species was a data point. All analyses were conducted using R version 3.1.0 and the R packages lme4 and nlme (Bates et al. 2013; Pinheiro et al. 2014; R Core Team 2014). Data is publicly available online (Shackleton et al. 2014).

## Results

**Fig. 1:**
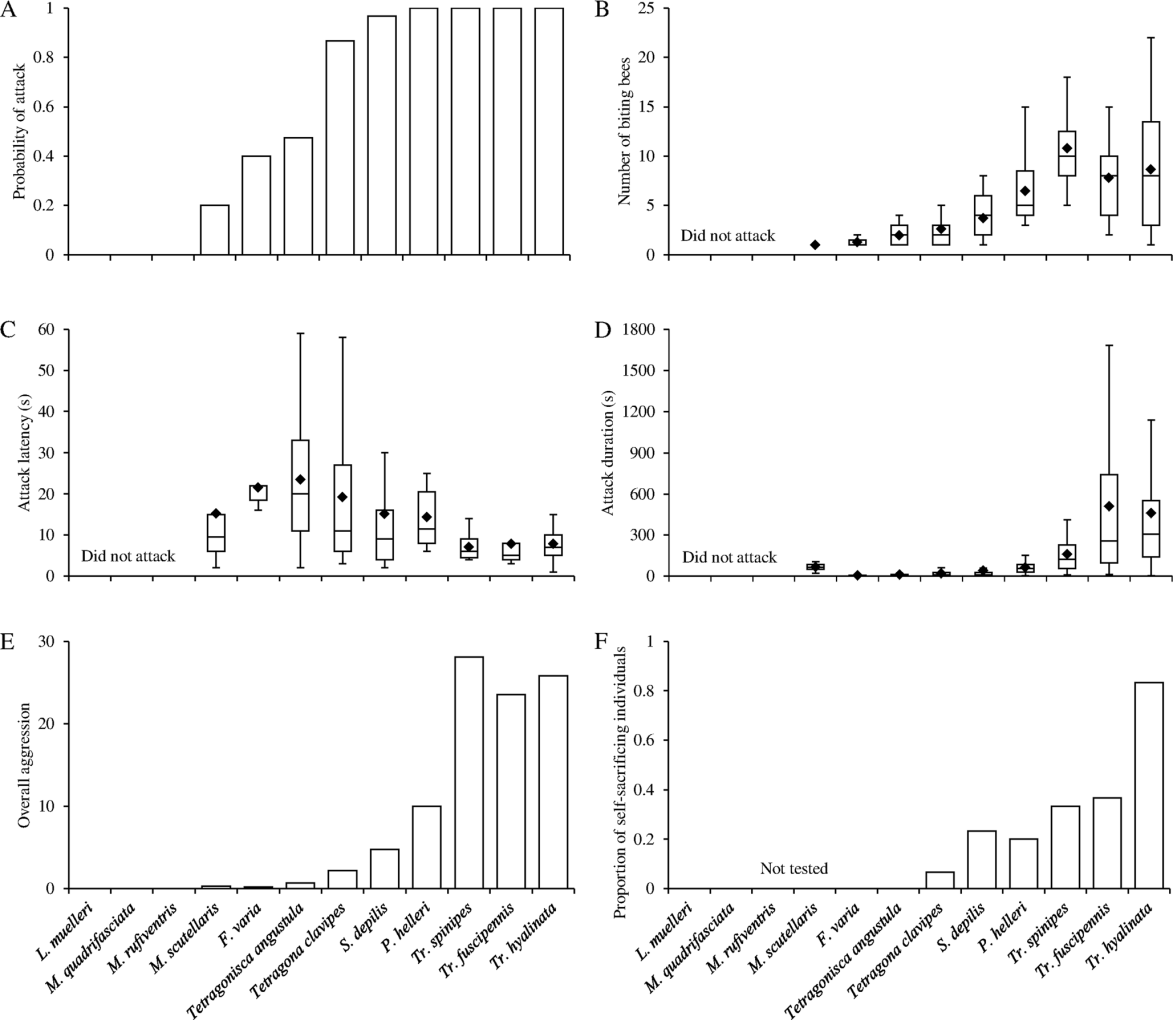
Variation in four measures of aggression in 12 stingless bee species in the flag test bioassay. **a** Probability of at least one bee from a colony biting the flag. **b** Number of biting bees per flag test. **c** Time until attacking the flag (latency). **d** Duration individual bees attacked the flag for. **e** Shows a combined measure of overall aggression. **f** The proportion of self-sacrificial individuals in the suicide bioassay. Whiskers 1.5 × interquartile range, means shown as *diamonds*

We studied a total of 59 colonies making 302 flag tests that resulted in 868 bees biting the flags. There was considerable variation between bee species in the levels of all measures of defensive behavior. Bee species differed significantly in their likelihood to attack the flag (*P* < 0.001, *χ* ^2^ = 114.47, *D.F*. = 11, Fig. 1a) and fell into three broad categories. Three species (*Leurotrigona muelleri*, *Melipona quadrifasciata*, and *Melipona rufiventris*) did not respond aggressively at all. Rather, the guards always stayed within the entrance and often retreated further inside when provoked by the flag. This indicated that guards perceived the flag as a potential threat but chose not to attack. Five had an intermediate response (e.g., *T. angustula*) where guards would usually leave the entrance when provoked but did not always attack the flag. Four species (all *Trigona* species and *Partamona helleri*) were extremely aggressive and always attacked the flag.

Of the nine species that did attack, the number of bees that bit the flag varied significantly among species (*P* < 0.001, *χ* ^2^ = 65.80, *D.F*. = 8, Fig. 1b) ranging from an average of 7.8 ± 1.1–10.8 ± 0.99 (means ± standard error) bees in the three *Trigona* species, with the maximum of 10.8 for *T. spinipes*, to 1 in *Melipona scutellaris*, a 10-fold difference. The maximum number in a single flag test was 22 bees from a *Trigona hyalinata* colony. The non-zero minimum of one bee occurred consistently in *M. scutellaris*. This species attacked the flag only 20 % of the time (Fig. 1a) but when it did, just one bee bit the flag (mean = 1 ± 0, Fig. 1b). This species’ nest has a narrow entrance hole that normally has a single guard present, blocking most of it (Couvillon et al. 2008a).

Species also varied significantly in the latency of attack with a three-fold difference (range = 7.0 ± 0.75–23.5 ± 4.0, *P* = 0.002, likelihood ratio = 24.06, *D.F*. = 8, Fig. 1c) and duration of attacks with over a 100-fold difference (range = 3.5 ± 1.1–508.7 ± 59.7, *P* < 0.001, likelihood ratio = 221.58, *D.F*. = 8, Fig. 1d). The three *Trigona* species attacked with the shortest latencies (7.0 ± 0.7–7.9 ± 1.9 s) and longest durations (157 ± 12.4–509 ± 59.7 s). The longest single bee attack duration was in *T. fuscipennis* at 51 min and 45 s. Figure 1e shows a combined overall aggression showing the more aggressive nature of *Trigona* versus the other species.

Of those species that did attack, all did so with mean latencies of <24 s, indicating that the flag waving period of 1 min was enough to provoke any colony likely to attack into attacking. Six species, *Tetragona clavipes*, *Scaptotrigona depilis*, *P. helleri*, *T. fuscipennis*, *T. hyalinata*, and *T. spinipes*, met the threshold of an attack probability >0.5 and mean attack duration >15 s and were used in the suicide bioassay.

In the suicide bioassay, the proportion of self-sacrificial individuals differed significantly with species (*P* < 0.001, *χ* ^2^ = 19.267, *D.F*. = 5, Fig. 1f), but all species that bit the flag had at least some individuals willing to suffer fatal damage rather than disengage. Suicidal individuals were observed to clamp their mandibles into the flag, and their refusal to relinquish their grip resulted in the fatal damage. *Trigona* spp. had the highest mortality, 33–83 %. *T. hyalinata* was especially suicidal with 83 % of individuals being pulled apart by the forceps rather than letting go. This was both over twice as high as the next highest species, *T. fuscipennis*, and was the only species where the proportion of suicidal individuals was greater than 50 %.

### Pain scale

**Fig. 2:**
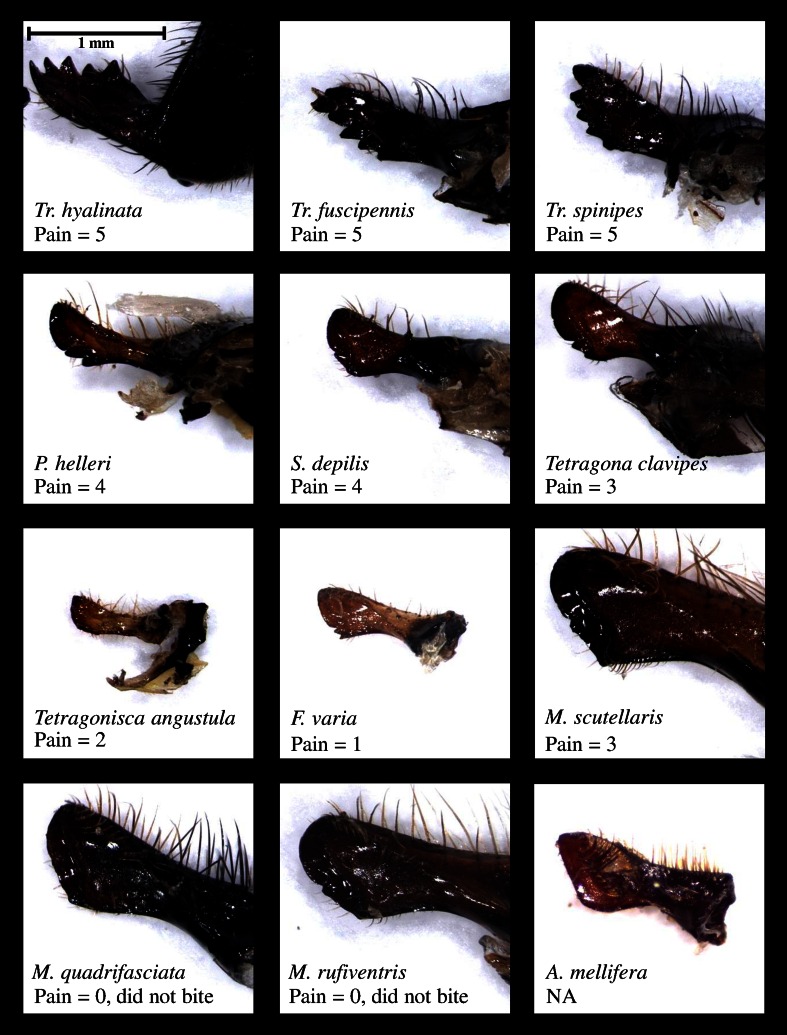
Mandibles from 11 stingless bee species and the honey bee (*Apis mellifera*) for comparison, showing the presence of teeth, particularly on *Trigona*. The pain caused by biting is shown on a scale of 0–5 where 0 = could not be induced to bite, 1 = biting was visible but could not pinch skin, 2 = able to pinch skin but caused no pain, 3 = very mild pain, 4 = moderate pain, and 5 = sharper unpleasant pain and capable of breaking skin if persistent. All pictures to same scale

In testing the pain different bee species caused from bites, three out of 12 species could not be provoked into biting. These were the same three species that could not be provoked into attacking during the flag tests (Fig. 1a). Of the bees which did bite, there was considerable variation in pain, but none compared in pain to the sting of a honey bee worker (Fig. 2). The *Trigona* species were the most painful of all. Overall aggression (Fig. 1e) was significantly and positively correlated with pain (Spearman’s rank, *P* < 0.001, *r* = 0.979, *n* = 12), indicating that the more aggressive species had the more painful bites.

The mandible photographs show that some species, particularly the three *Trigona*, have sharp teeth (Fig. 2). By comparison, the mandible of a worker honey bee *A. mellifera* is toothless and spoon-like in shape, a morphology shared by some of our study species such as the three *Melipona* species. Some species such as *T. clavipes*, which was moderately aggressive in our bioassays, had two small teeth on the basal part of each mandible which was otherwise spoon-shaped. All species of *Trigona* possessed serrated mandibles with five large teeth.

## Discussion

Our study shows that suicidal biting as an antipredator defense occurs in stingless bees. We believe this is the first clear demonstration of suicidal biting by any insect worker. The results support our general impression from casual observations that *Trigona* species are particularly defensive and even suicidal. The three *Trigona* species led the nine other species in all four aggression measures in the flag test, had the most painful bites, and had the largest proportion of self-sacrificial individuals in the suicide bioassay. The most suicidal was *T. hyalinata* in which almost all the bees tested (83 %) suffered fatal damage rather than disengage their mandibles from the flag. However, self-sacrifice was not confined to *Trigona* as it occurred in a significant proportion (7–23 %) of the test bees in the three other species submitted to the suicide bioassay (*P. helleri*, *S. depilis*, *T. clavipes*). This represents a new example of convergent evolution with other suicidal insect workers. Because levels of within-species self-sacrifice may be low, suicidal behavior may be difficult to detect and, therefore, potentially more widespread than previously thought. However, our results make it clear that not all stingless bees have suicidal biting. In fact, three of our study species never attacked the target flag at all.

For suicidal worker defense to evolve, the inclusive fitness benefits gained over non-suicidal defense, in terms of repelling intruders, must be greater than the costs incurred due to reduced worker numbers. Bees which attack more often, in greater numbers, with shorter latencies, and for longer durations will presumably be more effective at repelling the current attack and deterring potential future attacks (Schmidt 1990). Furthermore, in committing self-sacrifice through their jaw clamping behavior, stingless bees can immobilize or kill intruding insects (Grüter et al. 2012) and cause longer-lasting pain to vertebrate predators, preventing further attack on their colonies.

Higher levels of colony defense are likely to increase both colony survival and the mortality risk to the defender. Natural selection should, therefore, favor an optimal level of defense, where colony survival is traded off against the future value of the defender to the colony (Andersson et al. 1980). The optimal level of defense should increase with colony size because the colony contains more kin and is thus of greater value. In social insects, colony size can be large, 10,000s of individuals in *Trigona* for example (Roubik 2006), while the reproductive value of workers is low. Furthermore, many social insects, including stingless bees, exhibit age polyethism (Sommeijer 1984), where the risky tasks such as guarding are performed by the older workers with shorter life expectancies (Tofilski 2002). These factors can lead to a very high investment in defense and, potentially, the decision to commit self-sacrifice (de Catanzaro 1986; Brown et al. 1999). Eusocial insects could thus be described as having an exaptation for self-sacrificial behavior.

In social insects, each additional worker adds proportionally less to colony fitness (Michener 1964; Nonacs 1991). The relative costs of sacrificing workers will thus be less in large colonies than small ones. Stingless bee species vary greatly in colony size (Wille 1983). A suicidal attack of 20 bees from a 10,000-strong *Trigona* colony versus a 100-strong *Melipona* colony represents a loss of 0.2 versus 20 % of the total worker population. In small colonies, the costs of mass attacks, especially those involving suicidal behavior, would likely be greater than the benefits from improved defense. Our results support this theory, as the most aggressive and self-sacrificial species in the study (*Trigona*) were those with the largest colonies (Roubik 2006). Attacking intruders singly does not represent an effective defensive strategy because the pain and damage per bite, while unpleasant, is unlikely to drive an intruder away. Mass attacks are therefore required to offer a more robust defense. A formal analysis of colony size and aggression is not within the scope of this paper, as only 12 species were studied, of which suicidal behavior was only observed in six. This does, however, raise two important questions: First, is the mean colony size of a species a good predictor of aggression and self-sacrificial behavior? Second, is there intraspecific plasticity in aggression and self-sacrifice between small and large colonies?

Bites from the most aggressive species, namely the three *Trigona* species, were the most painful. The non-aggressive species which could not be provoked into attacking in the flag tests could not be provoked into biting human skin either. Larger species also tended to be more painful, but the only species to bite larger than the three *Trigona* species, *M. scutellaris*, was only mildly painful. Closer examination of the mandibles revealed that the *Trigona* species possessed serrated mandibles bearing sharp teeth. This morphological specialization presumably allows *Trigona* to cause more pain and damage to intruders, and as pain was correlated with overall aggression, it suggests that these mandibles are adaptations which enhance colony defense.

Mandibular teeth, however, may have also evolved in response to other selective pressures. Stingless bees use a variety of materials to construct their nests including resin and soil (Wille 1983). Toothed mandibles may aid in the acquisition and manipulation of such materials, as *Trigona* mandibles are similar in appearance to those of mason bees (e.g., *Osmia bicornis*: Megachilidae) and reminiscent of the fossorial forelegs of mole crickets (Gryllotalpidae). However, *P. helleri* nests are composed largely of soil but this species possesses only a single small tooth on each mandible. *Trigona* are also known to aggressively defend foraging patches against other bees, using their mandibles to harass, bite, and kill competitors (Johnson and Hubbell 1974; Nagamitsu and Inoue 1997). The vulture bee *Trigona hypogea* feeds on carrion and fruit in place of pollen and nectar, and mandibular teeth may facilitate foraging on such alternative food sources (Roubik 1982). While the *Trigona* species in the present study are not obligatory necrophagous, Wille (1983) suggested that they may turn to carrion when pollen sources are scarce.

Although the serrated mandibles of *Trigona* are a morphological feature that almost certainly enhances the effectiveness of their biting defense, their self-sacrifice is primarily behavioral in nature through simply refusing to let go. Bees in the suicide bioassay were often so engrossed in their attack on the flag that they made no attempt to evade the brush or forceps. Suicidal biting differs from most previously known examples of self-sacrifice in worker insects in lacking a morphological mechanism that guarantees mortality. Honey bee sting autotomy and autothysis in *Camponotus* spp. ants both nearly always result in the death of the worker (Hermann 1971; Shorter and Rueppell 2012). The stingless bees in our study, however, showed a gradation in suicidal behavior, and in all but *T. hyalinata*, mortality was less than 50 %.

Non-aggressive stingless bee species should not be thought of as defenseless, as biting is only one of a wide variety of defensive adaptations (Kerr and de Lello 1962). For example, when provoked during the flag tests, guards from the non-aggressive species in our study retreated from the nest entrance rather than confront the flag. These species tended to be those with very small entrances relative to their body sizes (Couvillon et al 2008a). This strategy represents the opposite of a mass attack, where intruders must combat guards singly in a narrow space. Several species in our study, most notably the mildly aggressive *Frieseomelitta varia* and moderately aggressive *T. clavipes*, frequently deposited sticky, odorous resins on the flag. While this behavior would have little effect on a vertebrate predator, it is likely very effective at immobilizing other stingless bees and may be similar in function to secretions found commonly in ants and termites (Prestwich 1979; Bordereau et al. 1997; Davidson et al. 2012).

Killing or disabling intruders is especially important in defending the nest against robbing by other stingless bees, as allowing scout robber bees to successfully scout can result in mass attacks on the colony and potentially far greater costs than the loss of a few suicidal workers. Biting defense is seen in the conflicts between one of our study species, *T. angustula*, and the obligate robber bee *Lestrimelitta limao*. Despite a large size disadvantage, *T. angustula* guards are able to clamp onto the wings of *L. limao* for long durations. This prevents the robber from flying and returning to its own colony but often results in the death of the *T. angustula* worker (Grüter et al. 2012). This is paralleled by the thermal defense displayed by honey bee workers against scouts of the Asian giant hornet *Vespa mandarinia* (Ono et al. 1995).

Our study has shown a wide range in the aggressive, defensive behavior of stingless bees. The presence of suicidal defensive biting in half our study species indicates that this behavior is potentially a widespread defensive strategy. In our experience, the three *Trigona* study species will almost invariably attack any human standing within a few meters of a nest entrance, often within seconds. Workers attack the head but also other parts of the body. So tenacious and unpleasant is the attack that the victim is forced into a hasty retreat. Bees are especially difficult to remove from hair, and if a bee is removed and released, it usually returns to the head immediately and resume its attack. The only recourse for the victim, therefore, is to flee and kill the bees to stop the attack.
